# Tribute to Dr Cherifa Sururu

**DOI:** 10.4102/phcfm.v13i1.2938

**Published:** 2021-04-14

**Authors:** Sunanda Ray

**Affiliations:** 1 Extraordinary Professor, Department of Medical Education, Faculty of Medicine, University of Botswana, Gaborone, Botswana


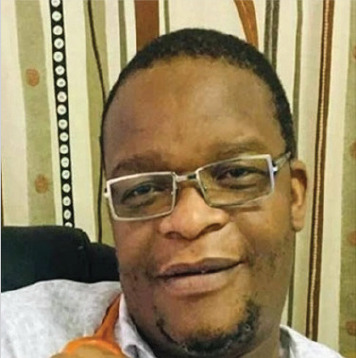


Family Physician Dr Cherifa Sururu died of Covid-19 related complications at Mater Dei Hospital, Bulawayo, on Wednesday 27 January 2021.

Cherifa was born on 18 March 1970 in Karoi district, Mashonaland West Province, Zimbabwe. His parents originally came from the Yao people of neighbouring Malawi, so he was brought up a Muslim, speaking Yao at home, but later became fluent also in Shona, Ndebele and English. He grew up on a commercial farm, one of 11 children, where his mother was a farm worker and the third wife of his father. His schooling in Karoi and later A-levels in Harare were made possible by a series of scholarships including from the Islamic Development Bank and other well-wishers. He contributed to the family income by joining with seasonal agricultural activities at commercial farms, and supplemented their food supplies with fishing and hunting.

Cherifa joined the University of Zimbabwe to study medicine and qualified with MBChB in 1997. He did his house jobs (internship) at Mpilo Central Hospital (1998–1999) and then embarked on a long career as a private general practitioner (GP) in Bulawayo. The entrepreneur spirit that saw him selling vegetables, sugar cane and wire toys as a young person, guided him in setting up several successful private surgeries in Bulawayo.

Cherifa was a founding member, trustee and second Chairperson of the Islamic Medical Association in Zimbabwe, over a 12-year period. He was active in the College of Primary Care Physicians of Zimbabwe (CPCPZ) where he was the National Treasurer 2006 to 2014 and Honorary National Secretary 2014 to 2017. He was also the CPCPZ representative for the Zimbabwe Medical Association (ZiMA) Matabeleland Branch for 2015 and 2016; Branch Vice President from 2016 to 2017; member of the Social Responsibility Committee from 2014 and organized several medical outreaches to underserved communities in districts. He represented the CPCPZ in the Medical & Dental Practitioners Council of Zimbabwe from July 2020 to his time of death. In the international arena he was the Zimbabwe country representative for Primafamed, a Primary Care and Family Medicine network in Africa, and through CPCPZ, a member of WONCA the World Organization of Family Doctors.

Cherifa was one of four private GPs who enrolled with the University of Stellenbosch Family Medicine MMed programme, with the ambition of setting up a similar programme in Zimbabwe. He gained his MMed in March 2017. A register of Specialist Family Practitioners was established at the Medical and Dental Practitioners Council of Zimbabwe in 2015 and the four pioneers of Family Medicine in Zimbabwe were the first to be entered into that register. Family Medicine MMed training programmes were established at the Faculty of Medicine, National University of Science and Technology (NUST), and at the University of Zimbabwe College of Health Sciences, which enrolled their first intakes in 2020 and for whom Cherifa was a committed educator.

Cherifa was involved in philanthropic work through Green Crescent Zimbabwe in partnership with the career guidance and counselling department of the Ministry of Primary and Secondary Education, to support a schools’ programme to prevent drugs and substance abuse, teenage pregnancies and suicide. The programme was launched in December 2020. At the time of his death, Cherifa was involved in research on factors influencing substance abuse among school children in Bulawayo and on quality of care in the Zimbabwe district health system.

Cherifa felt strongly connected to his parents’ country of origin and managed to visit Malawi in 2017 as part of a Training for Clinical Trainers in Family Medicine run by Stellenbosch University. As a child, he loved listening to the stories about Malawi told by his grandmother by the fireside at night.

His death is a sad loss to Family Medicine in Zimbabwe and he will be deeply missed. He is survived by his wife Elizabeth Chipendo and his five children.

